# P-2012. Predicting Persistent SARS-CoV-2 Shedding in Immunocompromised Patients: A Probability-Based Approach

**DOI:** 10.1093/ofid/ofae631.2169

**Published:** 2025-01-29

**Authors:** Eui Jin Chang, Jun-Won Kim, Choi-Young Jang, Sung Woon Kang, Ji Yeon Kim, Seongman Bae, Jiwon Jung, Min Jae Kim, Yong Pil Chong, Sang-Oh Lee, Sang-Ho Choi, Yang Soo Kim, Jeong-Sun Yang, Kyung-Chang Kim, Joo-Yeon Lee, Sung-Cheol Yun, Sung-Han Kim

**Affiliations:** Department of Internal Medicine, Asan Medical Center, Seoul, Korea, Seoul, Seoul-t'ukpyolsi, Republic of Korea; National Institute of Infectious Diseases, Cheong-ju, Ch'ungch'ong-bukto, Republic of Korea; Asan Medical Center, Seoul, Seoul-t'ukpyolsi, Republic of Korea; Asan medical center, Seoul, Seoul-t'ukpyolsi, Republic of Korea; Asan medical center, Seoul, Seoul-t'ukpyolsi, Republic of Korea; Asan Meidical Center, Songpa-gu, Seoul-t'ukpyolsi, Republic of Korea; Asan Medical Center, Seoul, Seoul-t'ukpyolsi, Republic of Korea; Asan Medical Center, Seoul, Seoul-t'ukpyolsi, Republic of Korea; Asan Medical Center, Seoul, Seoul-t'ukpyolsi, Republic of Korea; Asan Medical Center, Seoul, Seoul-t'ukpyolsi, Republic of Korea; Asan Medical Center, Seoul, Seoul-t'ukpyolsi, Republic of Korea; Asan Medical Center, Seoul, Seoul-t'ukpyolsi, Republic of Korea; National Institute of Infectious Diseases, Cheong-ju, Ch'ungch'ong-bukto, Republic of Korea; National Institute of Infectious Diseases, Cheong-ju, Ch'ungch'ong-bukto, Republic of Korea; Korea Disease Control and Prevention Agency, Cheongju, Ch'ungch'ong-bukto, Republic of Korea; Asan medical center, Seoul, Seoul-t'ukpyolsi, Republic of Korea; Asan medical center, Seoul, Seoul-t'ukpyolsi, Republic of Korea

## Abstract

**Background:**

Prolonged SARS-CoV-2 shedding frequently occurs in immunocompromised patients, making the determination of their isolation periods challenging. This study aims to estimate the probability of viral clearance in immunocompromised patients, stratified by elapsed days and other relevant variables.

Figure 1

**Figure:**
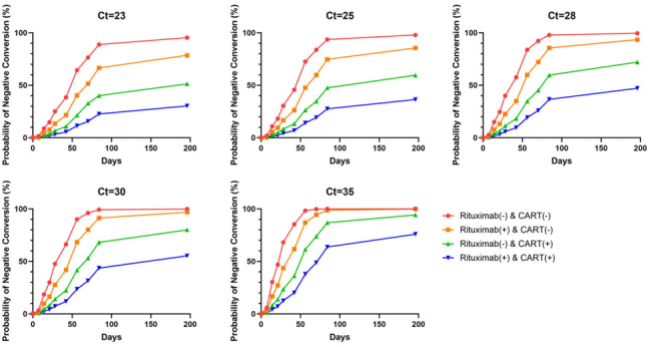

The estimated probability of viral clearance along days, stratified by SARS-CoV-2 PCR Ct value and immunocompromised conditions

**Methods:**

This prospective study was conducted at Asan Medical Center, a 2,732-bed tertiary teaching hospital in the Republic of Korea, from January 2022 to May 2023 during the prevalence of the Omicron variant. We enrolled immunocompromised patients, defined as 'moderately or severely immunocompromised' according to CDC guidelines. All participants, diagnosed with COVID-19 via nasopharyngeal swab PCR, provided weekly respiratory specimens for viral load measurement using real-time RT-PCR. Positive samples underwent viral culture. The Cox time-varying proportional hazard model was applied to identify significant predictors of viral culture negative conversion, employing backward elimination for model refinement. Analyses were conducted using R software, version 4.3.2, and included the assessment of viral copy number dynamics and treatment effects on viral shedding probabilities.

**Figure:**
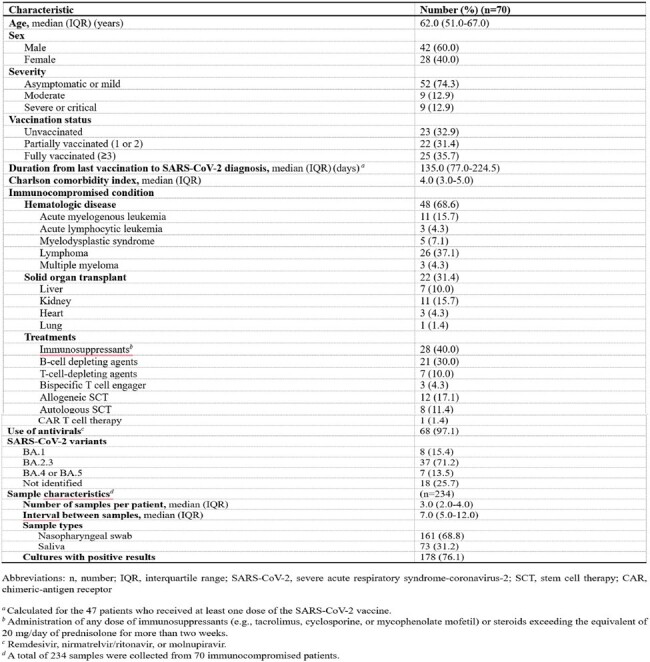

Baseline characteristics of the immunocompromised patients

**Results:**

In a cohort of 70 immunocompromised patients, 48 (68.6%) had hematologic malignancies and 22 (31.4%) underwent solid organ transplants. Univariate and multivariate analyses revealed that the use of B-cell depleting agents, CAR T-cell therapy, and viral copy number significantly influenced viral culture negative conversion. These findings were incorporated into a refined model to predict viral shedding probabilities. Patients with a history of both B-cell depleting agents and CAR T-cell therapy exhibited the lowest negative conversion rates, with median durations of viral shedding from 76 days to undeterminable depending on SARS-CoV-2 Ct values. In contrast, patients receiving neither treatment showed markedly faster viral clearance, typically within one to two months after COVID-19 diagnosis, regardless of Ct values.

**Figure:**
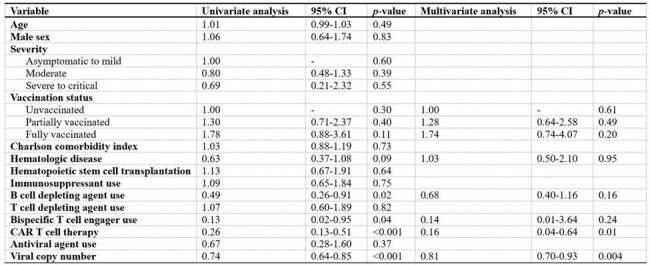

Predictive factors for viral culture negative conversion in the immunocompromised patients

**Conclusion:**

Ending the isolation of immunocompromised patients with COVID-19 should be determined individually, based on the viral copy number at specific time points and treatment conditions.

Table 3

**Figure:**
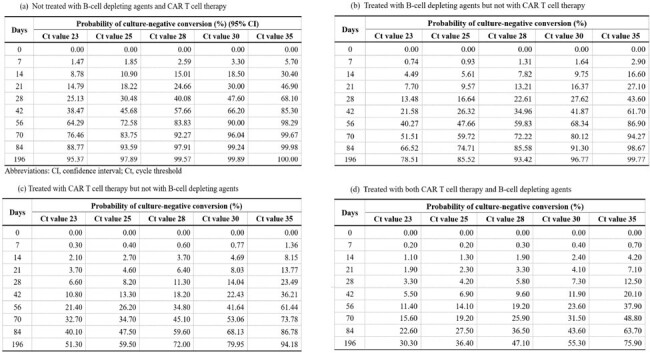

Probability of culture-negative conversion among immunocompromised patients based on treatments received, days elapsed after diagnosis, and SARS-CoV-2 PCR results

**Disclosures:**

All Authors: No reported disclosures

